# Supplementary material for: Association between oral microbiome and seven types of cancers in East Asian population: a two-sample Mendelian randomization analysis
Source: Front Mol Biosci. 2023 Nov 21;10:1327893. doi: 10.3389/fmolb.2023.1327893 (PMC10702768; doi:10.3389/fmolb.2023.1327893)
Supplement: Supplementary file 1 [file DataSheet1.ZIP › Supplementary Materials/MR plots of saliva/saliva═╝/ovarian cancer/pheno.2920_to_ovarian cancer_leave_one_out.pdf]

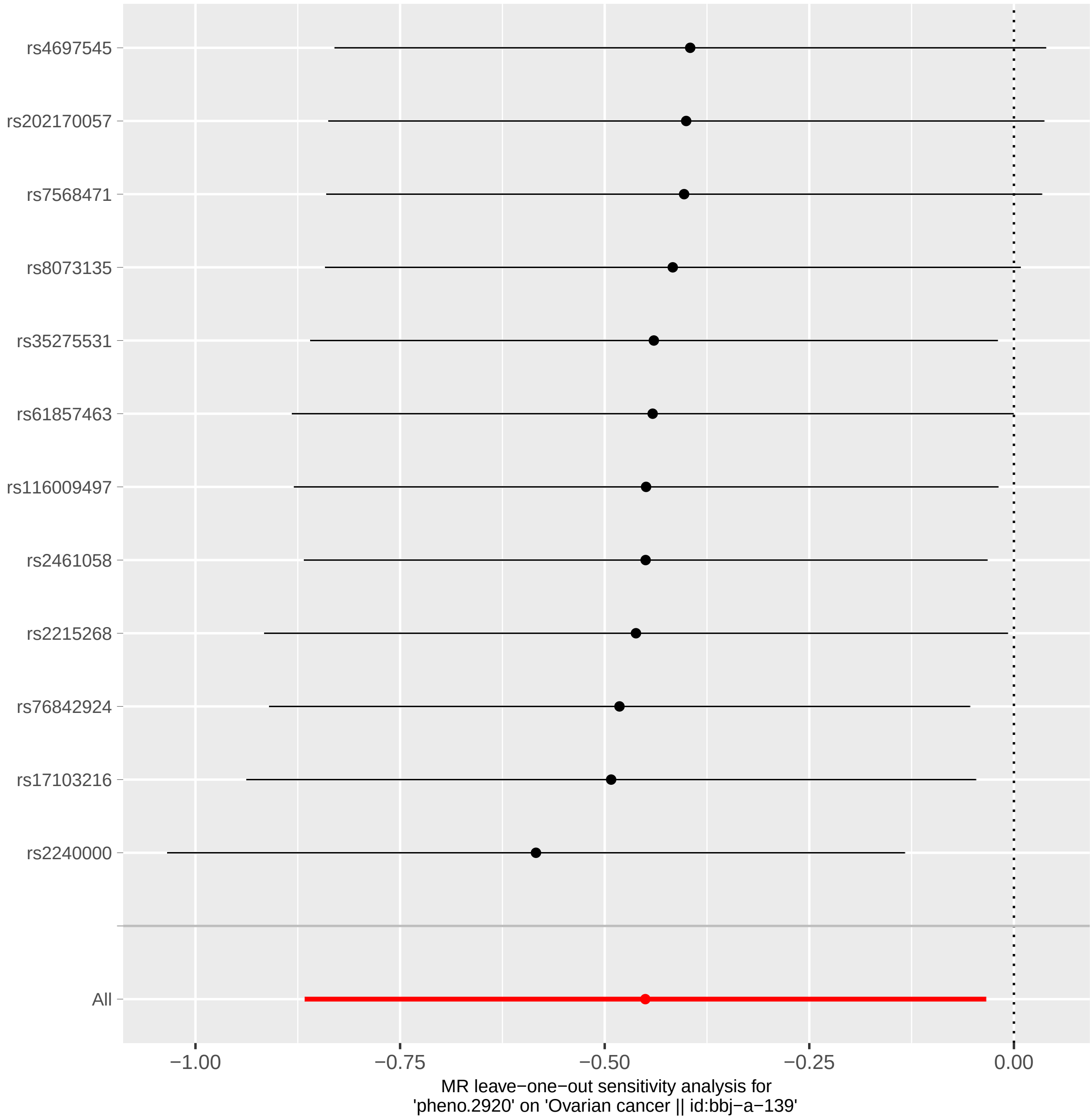

rs4697545
rs202170057
rs7568471
rs8073135
rs35275531
rs61857463
rs116009497
rs2461058
rs2215268
rs76842924
rs17103216
rs2240000
All
−1.00
−0.75
−0.50
−0.25
0.00
MR leave−one−out sensitivity analysis for
'pheno.2920' on 'Ovarian cancer || id:bbj−a−139'
